# Effects of ultraviolet-B radiation on physiology, immune function and survival is dependent on temperature: implications for amphibian declines

**DOI:** 10.1093/conphys/coaa002

**Published:** 2020-02-11

**Authors:** Niclas U Lundsgaard, Rebecca L Cramp, Craig E Franklin

**Affiliations:** School of Biological Sciences, The University of Queensland, Goddard Building (8), St Lucia, Queensland 4072, Australia; School of Biological Sciences, The University of Queensland, Goddard Building (8), St Lucia, Queensland 4072, Australia; School of Biological Sciences, The University of Queensland, Goddard Building (8), St Lucia, Queensland 4072, Australia

**Keywords:** chytridiomycosis, climate change, larvae, photolyase, synergistic interaction, thermal sensitivity

## Abstract

Multiple environmental changes are thought to be contributing to the widespread decline of amphibians in montane regions, but interactions between drivers of decline are not well understood. It has been proposed previously that elevated ultraviolet-B radiation (UBVR) and low temperatures may interact in their negative effects on health, immune function and disease susceptibility in exposed amphibians. In the present study, we chronically exposed larvae of the striped-marsh frog (*Limnodynastes peronii*) to a factorial combination of high and low UVBR and high and low temperature to assess interactive effects on growth, survival and indices of immune function. The high UVBR treatment reduced growth and survival of larvae compared to the low UVBR treatment at both temperatures, but the effects were significantly enhanced at low temperature. High UVBR exposure also induced a chronic inflammatory response as evidenced by an increase in the leucocyte proportion of total cells and altered the ratio of neutrophils to lymphocytes in the blood, highlighting a potential mechanistic basis for increased disease susceptibility in amphibians living at high altitudes. Our findings stress the importance of investigating environmental factors in combination when assessing their effects and highlight the mechanistic basis for how key environmental drivers in montane regions affect amphibian health. Continuation of this work is necessary for the development of targeted conservation strategies that tackle the root causes of montane amphibian declines.

## Introduction

Amphibians are at the forefront of the Anthropocene biodiversity crisis, with over 40% of extant species currently threatened with extinction (Stuart *et al*., 2004; IUCN, 2016). While habitat loss and overexploitation are well-known threats to amphibians, almost half of rapidly declining species are being affected by more elusive threats acting in relatively pristine, montane regions across much of the planet (Weygoldt, 1989; Laurance *et al*., 1996; Lips, 1998; Stuart *et al*., 2004). Although these declines at high altitude have been largely attributed to the emergence of a novel disease-causing chytrid fungus, *Batrachochytrium dendrobatidis* (*Bd*; Berger *et al*., 1998), widespread increases in ultraviolet-B radiation (UVBR; wavelength 280 to 315 nm) and changes in thermal regimes may have compounded the effects of this pathogen on affected species. These stressors are unlikely to act in isolation, instead forming complex interactive processes that may exacerbate declines (Carey, 1993; Kiesecker and Blaustein, 1995; Bancroft *et al*., 2008; Alton and Franklin, 2017). However, there is a lack of empirical research investigating such interactive effects, and data from studies exploring the potential link between elevated UVBR exposure and susceptibility to *Bd* infection have been conflicting or inconclusive (Garcia *et al*., 2006; Searle *et al*., 2010; Ortiz-Santaliestra *et al*., 2011).

UVBR is a potent natural stressor that forms pyrimidine dimers in DNA, which can subsequently lead to mutation, cancer and cell death (Mitchell and Nairn, 1989; Batista *et al*., 2009). UVBR also damages lipids, proteins and DNA indirectly by generating reactive oxygen species (ROS; Heck *et al*., 2003; Kazerouni *et al*., 2016a). Amphibians employ a suite of DNA repair mechanisms to remediate UVBR-induced DNA damage, including photolyase enzymes (Sancar and Sancar, 1988; Sancar and Tang, 1993; Schuch *et al*., 2015). However, widespread increases in UVBR resulting from the onset of stratospheric ozone depletion in the 1970s could be causing DNA damage at rates that can exceed the capacity for repair, especially in montane regions where ambient UVBR is already high (Farman *et al*., 1985; Kerr and McElroy, 1993; Herman *et al*., 1996; McKenzie *et al*., 1999). Indeed, studies often report lethal effects of ambient levels of solar UVBR for many amphibian species and at all stages of development (Blaustein *et al*., 1995; Tietge *et al*., 2001; Blaustein *et al*., 2005; Bancroft *et al*., 2008). UVBR can also interact with a range of natural and anthropogenic factors including pathogens, contaminants, aquatic pH and predatory cues to enhance the negative effects of UVBR on physiological performance and survival (see review by Alton and Franklin, 2017, and references therein). This is particularly relevant to amphibians in montane regions because low temperatures have been shown to exacerbate the negative effects of elevated UVBR on growth rates, development rates and swimming performance in amphibian embryos and larvae (van Uitregt *et al*., 2007). The potential interactive effects of UVBR and low temperature on amphibian immune function have received less attention.

UVBR-induced immunosuppression (photoimmunosuppression) is well documented in a range of organisms including fish (Jokinen *et al*., 2011), rodents (Kripke *et al*., 1992; Rivas and Ullrich, 1992) and humans (Poon *et al*., 2005). Three major mechanisms have been proposed by which UVBR exposure can induce immunosuppression in amphibians (Cramp and Franklin, 2018): (i) UVBR-induced DNA damage and oxidative stress in epidermal cells can directly trigger the release of immunosuppressive cytokines such as interleukin-4 and -10; (ii) the energetic cost of photoprotection and the repair and resynthesis of UVBR-induced molecular damage may reduce the allocation of energy towards other energetically expensive functions including immunity (Debecker *et al.,* 2015); and (iii) UVBR may activate the stress axis, which induces the release of glucocorticoids that, amongst other functions, are immunomodulatory (Dhabhar *et al*., 2010). Only a few studies have demonstrated the independent effects of elevated UVBR and low temperatures on immune function in developing amphibians, either through direct measures of immune system traits (Raffel *et al*., 2006; Ceccato *et al*., 2016), or through a disease challenge following UVBR exposure (Kiesecker and Blaustein, 1995; Ribas *et al*., 2009). The current study is the first empirical investigation of the interactive effects of elevated UVBR and temperature on larval amphibian immune function.

Our aim was to determine the independent and interactive effects of UVBR and temperature on striped-marsh frog tadpoles (*Limnodynastes peronii*, Dumeril and Bibron, 1841) at the gene, protein, cellular and organismal level. We hypothesized that simultaneous exposure to high UVBR and low temperature would result in reductions in survival, growth and developmental rates, increased antioxidant concentrations and an upregulation in photolyase and glucocorticoid receptor gene expression in larvae. These physiological responses are expected consequences of UVBR-induced molecular damage, reductions in energy availability and activation of the stress axis—mechanisms known to cause immunosuppression in other taxa. Hence, we hypothesized that immune function would be compromised and would be evidenced by reduced leucocyte proportion of total cells in larvae exposed to the high UVBR/low-temperature treatment combination.

## Methods

### Ethics statement

This research was approved by The University of Queensland Animal Ethics Committee (approval no. SBS/148/16/URG/ARC), and animal collection permission was granted by the Queensland Department of Environment and Heritage (permit no. WISP12218412).

### Study species

*L. peronii* is a common species that occurs across a wide thermal range along the eastern coast of Australia, from the tropics of northern Queensland down to Tasmania (16–42°S, Wilson and Franklin, 1999). Larvae of this species may be exposed to environmental temperatures ranging from 10 to 34°C during the course of their development (Wilson and Franklin, 1999), and they occur in regions where UVBR levels reach up to 500 μW cm^−2^ (in air at water level, van Uitregt *et al*., 2007). Eggs are laid in foam nests at the water’s surface and newly hatched larvae hang at the water surface for up to 2 days. Larvae have been observed to bask in sunny spots when water temperatures are low.

### Animal collection and experimental design

Eight *L. peronii* egg masses were collected the morning after laying from Sheep Station Creek in Morayfield, Australia (−27.105S, 152.952E), in an area with no forest canopy and exposed to sunlight. The eggs were transported in plastic bags containing creek water to The University of Queensland where they were maintained in plastic containers at 22.5 ± 1°C, under a 12L: 12D photoperiod regime using non-UVBR fluorescent lights, until commencement of experimental treatments. Larvae were fed thawed spinach *ad libitum* and water changes (carbon-filtered Brisbane city water) were made every second day to maintain water quality.

#### Experiment 1—effects of UV-temperature interactions on whole-animal and molecular physiology

Eight days after collection, larvae had developed to Gosner (1960) Stage 25 and a portion of these animals were randomly allocated to one of the four treatment combinations of low- and high-intensity UVBR (1.6 ± 1.2 and 24.4 ± 1.9 μW cm^−2^, respectively; see Table 1) with low and high temperatures (15.3 ± 0.5 and 25.9 ± 0.5°C, maintained throughout UVB irradiation). Each treatment had nine, 2-L plastic containers (17 × 17 × 9.5 cm), each holding 13 tadpoles in 1 L of filtered tap water at a depth of 5 cm (*n* = 117 larvae per treatment). Larvae were exposed to these treatments for 4 weeks. Containers were held in water baths (61 × 43 × 17 cm; six containers per water bath, six water baths in total) in a controlled temperature room set to maintain the low temperature treatment (15°C), with aquarium heaters added to the high-temperature treatment water baths (26°C). Water pumps were used to circulate and mix the water throughout the baths. To minimize the effect of an acute temperature change on the tadpoles during treatment allocation, the original housing temperature (22.5°C) was changed progressively over a 48-h period (prior to UVBR exposures) via adjustments to room temperature and aquarium heaters until the specified treatment temperatures were reached.

**Table 1:** Ultraviolet-A (UVA) and ultraviolet-B (UVB) irradiance levels (μW cm^−2^) and absolute dose estimates (kJ m^−2^) of the four treatments for each experiment

	**High UV**		**Low UV**	
	**15°C**	**26°C**	**15°C**	**26°C**
**UVA irradiance (μW cm** ^**−2**^ **)**	101.1 ± 6.1	99.4 ± 6.9	45.2 ± 7.8	44.0 ± 8.2
**UVA absolute dose estimate (kJ m** ^**−2**^ **) for Experiment 1**	305.72	300.58	136.68	133.05
**UVA absolute dose estimate (kJ m** ^**−2**^ **) for Experiment 2**	203.81	200.39	91.12	88.70
**UVB irradiance (μW cm** ^**−2**^ **)**	24.8 ± 2.0	24.1 ± 1.9	1.9 ± 1.3	1.4 ± 1.0
**UVB absolute dose estimate (kJ m** ^**−2**^ **) for Experiment 1**	74.99	72.88	5.75	4.23
**UVB absolute dose estimate (kJ m** ^**−2**^ **) for Experiment 2**	50.00	48.58	3.83	2.82

Differences in absolute dose values between experiments resulted from *L. peronii* larvae in Experiment 2 being exposed for 2 h per day for 4 weeks, as opposed to larvae in Experiment 1 which received 4 h of exposure per day for the first 2 weeks. Measurements were taken using a radiometer/photometer (IL1400BL, International Light Inc., Newburyport, USA) at the water surface of each of the nine replicate containers per treatment. Irradiance presented as mean ± standard deviation.

Survival was assessed daily, and the size (total length and body width; see Measures section) of remaining larvae were recorded at Weeks 0, 2 and 4 of the exposure period. The oxygen consumption rates of tadpoles were measured using closed system respirometry at the end of the 4-week exposure period, after which they were euthanized in buffered Tricaine-S (MS-222; Aqua-Life, Nanaimo, Canada; 0.5 g L^−1^; all subsequent euthanasia was conducted using this method) for measurements of total antioxidant capacity and gene expression (see Measures section).

**Figure 1:**
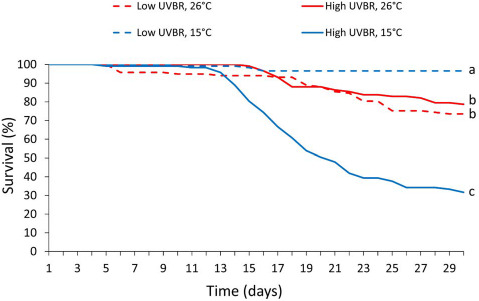
Effect of high (solid lines) and low (dashed lines) UVBR in combination with either low (blue lines) or high (red lines) temperature, on the percentage survival of *L. peronii* larvae (*n* = 117 per treatment; Experiment 1). Lines show the trend in mortality for each treatment over the 30-day exposure period. Letters denote significant differences between treatment groups

The UVBR conditions were generated in addition to the 12L: 12D background room lighting regime (non-UV) using six 40-W fluorescent light sources (Repti-Glo 10.0, Exo Terra, Montreal, Canada) mounted ~70 cm above containers. A UVBR-blocking film (Melinex/Mylar 516 100 μm, Archival Survival, Victoria, Australia) was suspended 47 cm above the low UVBR treatment containers to reduce UVBR levels in this treatment. UVBR and ultraviolet-A (UVAR) irradiance levels were measured using a radiometer/photometer (IL1400BL, International Light Inc., Newburyport, USA) at the water surface of each container to ensure consistent UVBR levels (Table 1). Tadpoles were exposed to a 4-h pulse of UVBR during the daily midpoint (1000–1400) for the first 2 weeks, which was reduced to 2 h (1100–1300) per day for the last 2 weeks due to unexpectedly high mortality rates during the first half of the experimental period in the high UVBR treatments (Fig. 1). The cumulative dose of UVBR was ~74 kJ m^−2^ (daily exposure of 1.75–3.5 kJ m^−2^) for the high UVBR treatments and 5 kJ m^−2^ (daily exposure of 0.12–0.24 kJ m^−2^) for the low UVBR treatments, dispersed over the 4-week exposure period (for exact values, see Table 1).

The UVBR and temperature conditions of the experimental treatments were intended to reflect the natural conditions where the animals were sampled. Due to the complex temporal and spatial variability of UVBR in natural settings, we did not quantify the exposure received by larvae in the field. To ensure that effects were not overestimated in the present study, UVBR levels used are much lower than ambient UVBR levels measured in the region (20 times lower than levels measured in air at water level in Brisbane, Australia; van Uitregt *et al*., 2007). Our conservative UVBR treatments account for attenuation expected from cloud cover, vegetation cover and dissolved organic matter concentrations in the water at the collection site (Crump *et al*., 1999; Palen *et al*., 2002; Trenham and Diamond, 2005; Palen and Schindler, 2010; Alton and Franklin, 2017; but see also Blaustein *et al*., 2004). The 2–4-h daily UVBR exposure period is also ecologically realistic given that the period around solar zenith (midday) likely represents the period where UVBR exposures are greatest.

#### Experiment 2—effects of sub-lethal UV-temperature interactions on immune function

Upon completion of the first experiment, additional tadpoles (from the clutches collected for Experiment 1) that had been raised under control conditions (22.5 ± 1°C, 12L: 12D photoperiod, non-UV) were used in Experiment 2. Unlike Experiment 1, these larvae were 5 weeks old at the commencement of treatment exposures (Gosner stage 25–26) and UVBR exposure was reduced to 2 h per day in an attempt to improve survival. This exposure regime reduced the cumulative dose of UVBR to ~49 kJ m^−2^ (daily exposure of 1.75 kJ m^−2^) for the high UVBR treatments and 3.3 kJ m^−2^ (daily exposure of 0.12 kJ m^−2^) for the low UVBR treatments. Individual tadpoles were kept separate in 330-mL plastic cups filled with 250 mL filtered water (5 cm water depth), with four cups placed in each of the plastic containers from Experiment 1 (*n* = 36 animals per treatment). Larvae were maintained under these conditions for 4 weeks. Size and developmental stage of tadpoles were recorded; however, only developmental rate is presented here for Experiment 2 (for Experiment 2 growth results, see Supplementary Fig. 1). At the end of the 4-week exposure, the animals were euthanized and blood samples were collected from a random selection of the animals for leucocyte counts (see Measures section).

### Measures

#### Growth and development

Body size metrics were collected from a random selection of six tadpoles per container (*n* = 54 per treatment). Body size was measured as total length (snout to tip of tail) and body width (widest point) every 2 weeks. Tadpoles were placed individually in a petri dish and photographed (iPod touch fifth generation, Apple, CA, USA) from a dorsal viewpoint on 1-mm-gridline paper. Dimensions were taken from these photographs using the imaging software ImageJ (National Institutes of Health, Bethesda, MD, USA), and growth was calculated by subtracting the initial size at Week 0 from subsequent size measures, to get the change in size over the 4-week exposure period. The Gosner developmental stage (Gosner, 1960) of each tadpole in Experiment 2 was determined at the commencement and termination of the experiment.

#### Metabolic rate

After 4 weeks of UVBR-temperature exposures, the oxygen consumption rate of 18 tadpoles from each treatment in Experiment 1 was measured using closed system respirometry. Individual tadpoles (fasted for 48 h) were placed in separate 25-mL syringes each fitted with an oxygen sensitive sensor spot (PreSens, Regensburg, Germany), filled with air-saturated water and sealed with a three-way tap. Following a 30-min adjustment period, aquatic oxygen concentration measures (% air saturation) were made non-invasively through the syringes at 0 min, 30 min and 1 h using a Fibox System (PreSens GmbH, Fibox 3, Germany) to determine the rate of change in oxygen concentration resulting from larval respiration. Tadpoles from the high-temperature treatment were contained in syringes floated in a water bath set to 26°C to maintain temperature. All tests were conducted within a few hours of the middle of the day to minimize the effect of circadian variability on metabolism. The volume of water in each syringe was adjusted to achieve a sufficient drop in oxygen concentration within the hour. These volumes varied from 2 to 20 mL depending on the treatment, and this was accounted for in the equation for rate of oxygen consumption (VO_2_: mL O_2_ h^−1^):

$$ \textrm{VO}_2= - 1 \times ([m_{a}- m_{c}]/100)\times \textrm{V} \times \beta \textrm{o}_{2} $$

where *m*_a_ is the rate of change of oxygen saturation for a respirometry chamber containing a tadpole (% air saturation per hour), *m*_c_ is the background rate of respiration (respirometry chamber containing only water; % air saturation per hour), *V* is the volume of water in the respirometry chamber minus the volume of the animal (mL; assuming a tissue density of 1 g mL^−1^) and *β*o_2_ is the oxygen capacitance of water at each temperature (7.12 mL O_2_ L^−1^ at 15°C and 5.69 mL O_2_ L^−1^ at 26°C; Cameron, 1986). After oxygen consumption measures had been taken, larvae were euthanized and weighed.

#### Total antioxidant capacity

Total non-enzymatic antioxidant capacity (TAC) is an indicator of the ability of an organism to offset oxidative damage and is expected to increase when oxidative stress increases (Sies, 1997). A subset of tadpoles from each treatment in Experiment 1 were randomly sampled at Weeks 2 (*n* = 9 per treatment) and 4 (*n* = 7 per treatment) of exposure and were euthanized and stored at −80°C. Larvae were homogenized in 10 volumes of ice-cold PBS (0.01 M) for 30 s using a T25 Ultra-Turrax (IKA, Wilmington, NC, USA) and then centrifuged at 3000×g for 3 min at 4°C. TAC was determined from 10 μL of supernatant per sample using a commercially available total antioxidant capacity kit (MAK187, Sigma-Aldrich, Castle Hill, Australia), according to the manufacturer’s protocol. A Trolox standard curve (range 0–20 nmol Trolox; *R*^2^ = 0.9994) was prepared using standards supplied with the assay kit. The assays were placed on an orbital shaker and incubated in darkness for 90 min. Absorbance was measured at 570 nm using a multi-well plate reader (Beckman Coulter, DTX880, California, USA) with all reactions run in duplicate and at room temperature (25 ± 1°C). Absorbance values were converted to ‘Trolox equivalents’ using the standard curve. Trolox equivalents values were then divided by the sample volume (μL) to determine TAC (nmol μl^−1^). Final values were expressed in nmol mg^−1^ tissue by dividing the TAC value by 10 to account for dilutions.

#### Gene expression analysis of CPD photolyase, 6-4PP and glucocorticoid receptor

Six tadpoles per treatment from Experiment 1 were collected at Weeks 2 and 4 of exposure and were euthanized and stored at −20°C in RNAlater (Ambion, USA). Due to the small size of the animals, total RNA was extracted using a commercially available kit according to the manufacturer’s protocol (RNeasy Mini Kit, Qiagen, Hilden, Germany). Briefly, tissues were homogenized in RNeasy lysis buffer using a TissueLyser II (Qiagen) and 5-mm stainless steel beads. Homogenates were centrifuged to precipitate insoluble material, and RNA was isolated using a silica spin column. Genomic DNA was digested during the RNA extraction procedure using an on-column DNAse (Qiagen), and purified RNA was eluted into ultrapure water. The RNA concentration was quantified using a Qubit RNA assay kit (Thermo Fisher Scientific, MA, USA). Using a SensiFAST cDNA Synthesis Kit (Qiagen), 1 μg of total RNA from each sample was converted into cDNA with a no reverse transcriptase control included for each sample (no DNA contamination was detected). cDNA samples were then treated with RNAse H (Thermo Fisher) to remove any remaining RNA and purified using a QIAquick purification kit (Qiagen).

Species-specific primers (Table 2) against photolyase and glucocorticoid receptor gene transcripts were designed from an in-house larval *L. peronii* transcriptome. *Xenopus tropicalis* and *Xenopus laevis* reference DNA sequences (Table 2) were compared with the *L. peronii* transcriptome to identify highly similar sequences. Putative transcripts were compared with homologous sequences from other taxa using the nucleotide basic local alignment search tool (BLASTn) from the National Centre for Biotechnology Information (NCBI) database. Quantitative PCR (qPCR) primers against the target genes were designed using PrimerQuest (Integrated DNA Technologies, IA, USA) with acceptance of the default parameters. qPCR was performed using Power SYBR Green PCR Master Mix (Thermo Fisher Scientific) and the cycling parameters recommended by the manufacturer. Each assay (in triplicate) included a no-template control and a no reverse transcriptase control. All PCR efficiencies were >90%, and all assays produced unique dissociation curves. Data were collected using Bio-Rad CXF Manager software (version 3.1, Bio-Rad), and results were exported to Excel. Each gene was quantified relative to the expression of a housekeeping gene, β-actin, using the Pfaffl (2001) method. Expression levels were presented as fold change relative to average expression levels in larvae of the low UVBR, high-temperature treatment.

**Table 2:** : Species-specific primers used for qPCR analysis of CDP and 6-4 photolyase, glucocorticoid receptor protein and Beta-actin (housekeeping) genes in *Limnodynastes peronii* larvae

Gene Id	Accession number (reference sequence)	*Limnodynastes peronii* primers	Amplicon size (bp)
CPD photolyase (Class II CDP *phr*)	NP_001089127 CPD photolyase-like L homolog [*Xenopus laevis*]	F: CCT TGG GCG TAG TGT ATT G R: AGC CAA AGT GAC GGA TTG	159
6-4 photolyase (6-4 *phr*)	NP_001081421–6-4 photolyase [*Xenopus laevis*]	F: CAA ACT GTC CTG GCA TCT ATC R: TGT CCC AGC TCC TCT AAT G	140
Glucocorticoid receptor protein (*nr3c1*)	ENA_CAA51010.1 glucocorticoid receptor protein [*Xenopus laevis*]	F: CCC TCT TCT CAA ATG GCT ATG R: GTT GTG CTG ACC TTC TAC TG	164
Beta-actin (β-actin)	*Xenopus laevis* actin, beta L homeolog (actb.L), mRNA	F: TGC GTG ACA TCA AGG AGA AG R: CAA GGA ATG AAG GCT GGA AG	177

#### Leucocyte profiles

Blood samples were taken immediately after the 4-week treatment exposure period. Larvae were euthanized, and blood was extracted from the severed tail using a 75-μL heparinized hematocrit tube (Kimble Micro-Hematocrit Capillary Tubes, Rockwood, USA). The blood was smeared onto a glass slide and stained with Wright-Giemsa stain (ProSciTech, Thuringowa Central, Australia). Slides were viewed using a Nikon Upright Microscope (Eclipse Ci-S/Ci-L). Five frames of each blood sample (200–400 × magnification) were randomly selected across the feathered edge of the blood smear and photographed using a Nikon Digital Site DS-Fi1c camera. These images were analysed using the cell counter tool on ImageJ. Cells were counted from the centre of the image. Leucocyte numbers were recorded as a proportion of total cells (red and white) by counting 40 cells on each of five frames. The neutrophil-to-lymphocyte (N:L) ratio was calculated as the number of neutrophils divided by the number of lymphocytes in a sample of 100 leucocytes. Monocytes, basophils and eosinophils were scarce and difficult to identify and so were not assessed.

### Statistical analysis

All statistical analyses were performed using R version 3.4.2 (R Core Team, 2017). All data are presented as means ± standard error, unless otherwise stated. Survival data were modelled with a Cox mixed effects model with container ID as a random effect, utilizing the *coxme* (Therneau, 2018), *Matrix* (Bates and Maechler, 2017) and *survival* (Therneau and Grambsch, 2000; Therneau, 2015) packages.

Oxygen consumption measures were square root transformed. Oxygen consumption, growth and TAC were analysed with linear mixed effects models with container ID as a random effect, utilizing the *lmerTest* package (function *lme*; Kuznetsova *et al*., 2017). Growth was analysed as treatment-specific differences in body size at the end of the exposure period (Week 4), with initial body size (Week 0) as a random effect. Body mass was included as a covariate in models of TAC and oxygen consumption.

Differences between treatments in developmental stage after the 4-week exposure period were assessed with a mixed effects model with initial developmental stage as a random effect (animals were housed individually for Experiment 2, so no container effect was present for any variables assessed in Experiment 2). Leucocyte proportion of total cells and N:L ratios were analysed with binomial generalized linear models with developmental stage as a covariate, utilizing the *lme4* package (function *glm*; Bates *et al*., 2015).

mRNA expressions of larval photolyase and glucocorticoid receptor genes (*gene*) were compared between treatments (*UV* and *temp*) with the following linear mixed effects model according to Yuan *et al*. (2006):

$$\begin{align*} {E}^{-\Delta \Delta{C}_T}&={\beta}_0+{\beta}_{\mathrm{UV}}+{\beta}_{\mathrm{temp}}+{\beta}_{\mathrm{gene}}+{\beta}_{\mathrm{UV}}{X}_{\mathrm{temp}}\\ &\quad +{\beta}_{\mathrm{UV}}{X}_{\mathrm{gene}}+{\beta}_{\mathrm{gene}}{X}_{\mathrm{temp}}+{\beta}_{\mathrm{UV}}{X}_{\mathrm{temp}}{X}_{\mathrm{gene}}\\ &\quad +{b}_{\mathrm{ID}}+\epsilon \end{align*}$$

where ${E}^{-\Delta \Delta{C}_T}$ is the ΔΔ threshold cycle (C_T_) and $\beta$ is the fixed effects parameters (where 0 is the intercept and $X$ is the interaction term). ${b}_{\mathrm{ID}}$ is the random intercept for tadpole ID expressed as ${b}_{\mathrm{ID}}\sim N(o,{\delta}^2)$ (where ${\delta}^2$ is the variance of random intercept), and $\epsilon$ is the Gaussian error term expressed as $\epsilon \sim N(o,{\sigma}^2)$ (where ${\sigma}^2$ is the variance of residual).

Interactive effects of UVBR and temperature on gene expression were assessed post hoc by utilizing the function *testInteractions* from the package *phia* (*P* value adjusted using the Holm method; Rosario-Martinez, 2015). Tukey tests were used post hoc to assess interactive effects on survival, oxygen consumption rates and leucocyte counts using the *multcomp* package (function *glht*; Hothorn *et al*., 2008), as well as on growth using the *tukey.test* function (95% family-wise confidence level).

## Results

### Growth, development & mortality

There was a significant interaction between UVBR and temperature on mortality of *L. peronii* larvae over the 4-week exposure period (*Z* = −5.41, *P* < 0.001; Fig. 1). The level of UVBR exposure had no significant effect on survival at 26°C (GLHT, *Z* = 0.202, *P* = 0.997). At 15°C, however, mortality was 20 times greater in the high UVBR treatment compared to the low UVBR treatment, with 68.4% mortality after 4 weeks (GLHT, *Z* = −6.384, *P* < 0.001). Unexpectedly, under low UVBR exposure, larvae raised at 26°C experienced significantly higher mortality compared to larvae in the 15°C treatment (GLHT, *Z* = −3.318, *P* = 0.005).

**Figure 2:**
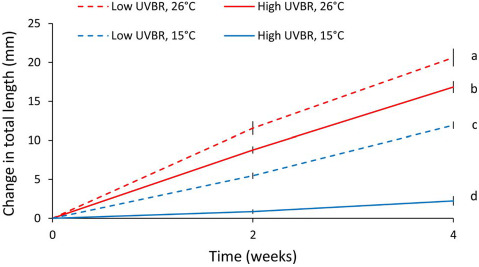
Effect of high (solid lines) and low (dashed lines) UVBR in combination with either low (blue lines) or high (red lines) temperature on the growth (as a change in total length) of a random subset of *L. peronii* larvae over the 4-week exposure period (*n* = 21–54; Experiment 1). Data are presented as means ± standard error at two time points (Weeks 2 and 4). Letters denote significant differences between treatment groups (same for Weeks 2 and 4)

High UVBR and low temperature interacted in their negative effects on growth of larvae (total length: *F*_1,30_ = 10.43, *P* = 0.003, body width: *F*_1,30_ = 18.93, *P* = 0.001; Fig. 2). At 26°C, high UVBR reduced growth in total length of larvae by 18.4% relative to low UVBR (Tukey HSD, *P* = 0.002). At 15°C, however, the effect of high UVBR was exacerbated, such that growth in total length of larvae was reduced by 81.3% in the high UVBR, 15°C treatment compared to growth of larvae in the low UVBR, 15°C treatment (Tukey HSD, *P* < 0.001).

**Figure 3:**
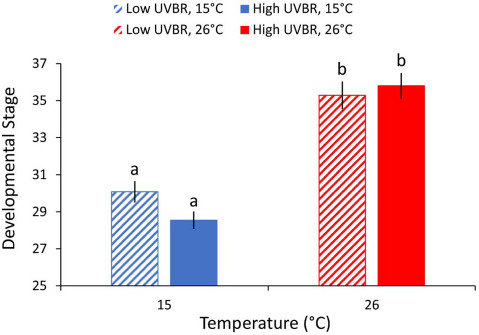
Effect of high (solid bars) and low (patterned bars) UVBR in combination with either low (blue bars) or high (red bars) temperature on the developmental stage (Gosner, 1960) of *L. peronii* larvae after 4 weeks of exposure to experimental treatments (*n* = 31–35 per treatment; Experiment 2). All larvae were at Gosner developmental stage 25 at the commencement of the exposure period. Data are presented as means ± standard error, and letters denote significant differences between treatment groups

Low temperature significantly reduced developmental rate of larvae (*F*_1,129_ = 97.085, *P* < 0.001), but no effect of elevated UVBR on development rate was detected (*F*_1,129_ = 1.151, *P* = 0.285; Fig. 3).

### Metabolic rate

**Figure 4:**
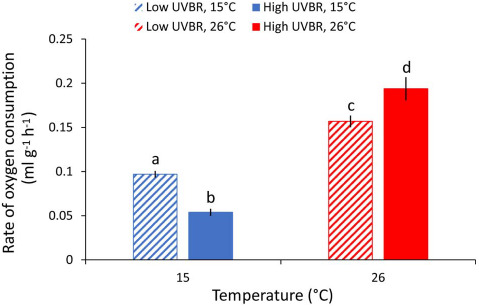
Effect of high (solid bars) and low (patterned bars) UVBR in combination with either low (blue bars) or high (red bars) temperature on the mass specific rate of oxygen consumption (mL h^−1^ g^−1^) of *L. peronii* larvae (*n* = 17 to 18 per treatment; Experiment 1). Data are presented as means ± standard error, and letters denote significant differences between treatment groups

As body mass and temperature increased, oxygen consumption rates increased (mass: *F*_1,64_ = 237.635, *P* < 0.001, temperature: *F*_1,64_ = 499.748, *P* < 0.001). There was a significant antagonistic interaction between UVBR and temperature on oxygen consumption rate (*F*_1,64_ = 18.101, *P* < 0.001); at 26°C, larvae exposed to elevated UVBR had higher oxygen consumption rates than larvae exposed to low UVBR (GLHT, *Z* = −2.755, *P* = 0.029), but larvae in the 15°C treatment had reduced oxygen consumption rates when exposed to elevated UVBR compared to exposure at low UVBR (GLHT, *Z* = 3.028, *P* = 0.013; Fig. 4).

### Total antioxidant capacity

**Figure 5:**
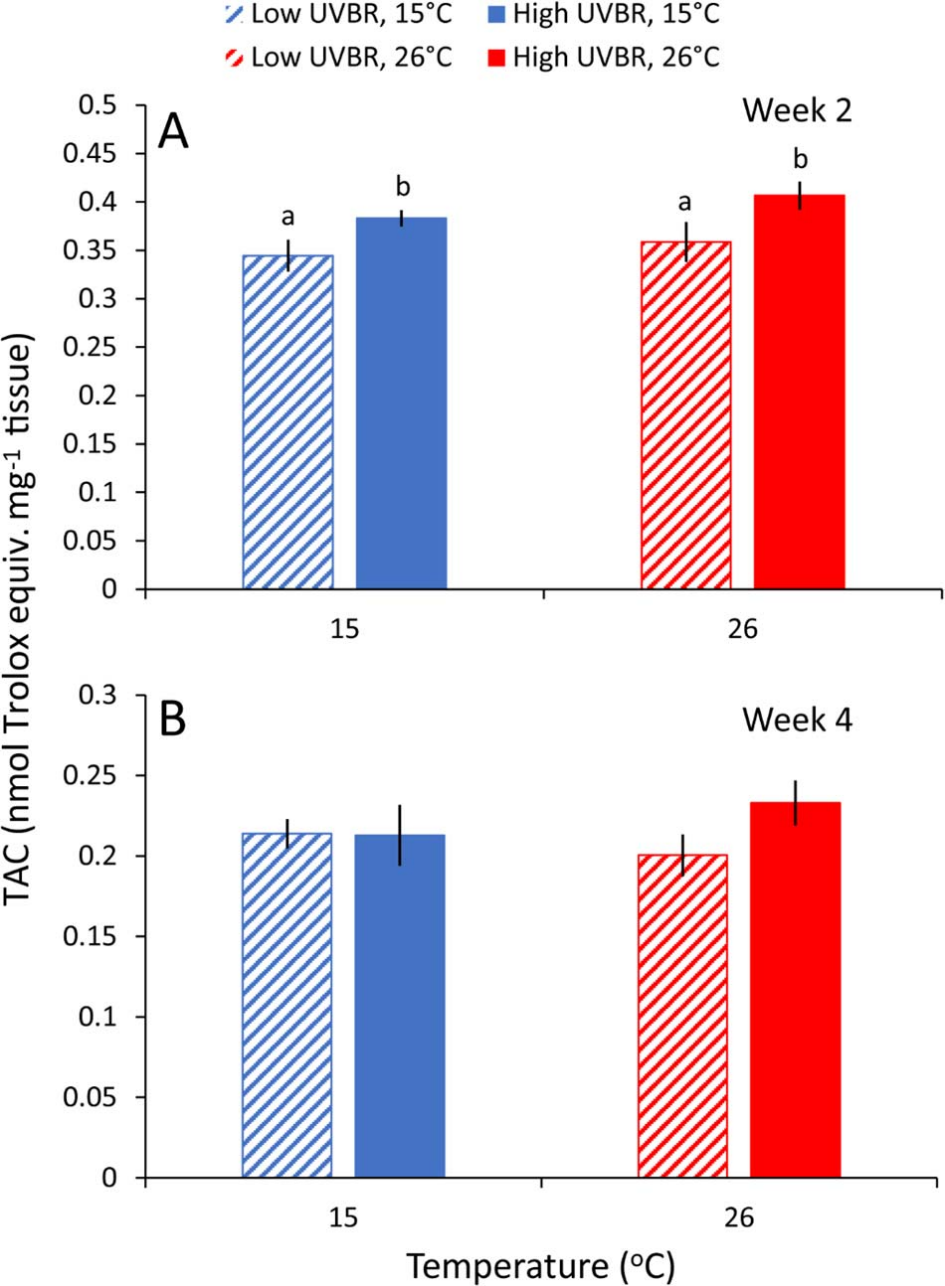
Effect of 2 (**A**) and 4 (**B**) weeks of exposure to high (solid bars) and low (patterned bars) UVBR at either 15°C (blue bars) or 26°C (red bars) on the total antioxidant capacity (TAC; nmol Trolox equiv. mg^−1^ of tissue) of whole-body *L. peronii* larva tissue (*n* = 6–9 per treatment; Experiment 1). Data are presented as means ± standard error. Lowercase letters above bars denote significant differences between treatment groups. Where no letters are presented, there were no significant differences between treatments

There was no effect of temperature on TAC after 2 and 4 weeks of larval exposure (*F*_1,30_ = 1.663, *P* = 0.207 and *F*_1,23_ = 0.061, *P* = 0.808, respectively; Fig. 5). However, larvae had an 11–13% increase in TAC after 2 weeks of exposure to high UVBR (*F*_1,30_ = 8.754, *P* = 0.006; Fig. 5A), but this elevated TAC was not apparent at the 4-week exposure mark (*F*_1,23_ = 1.311, *P* = 0.264; Fig. 5B). There was no significant interaction between temperature and UVBR (*F*_4,28_ = 3.116, *P* = 0.549), nor was there any effect of body mass on TAC (*F*_3,29_ = 4.122, *P* = 0.202).

### Gene expression

**Figure 6:**
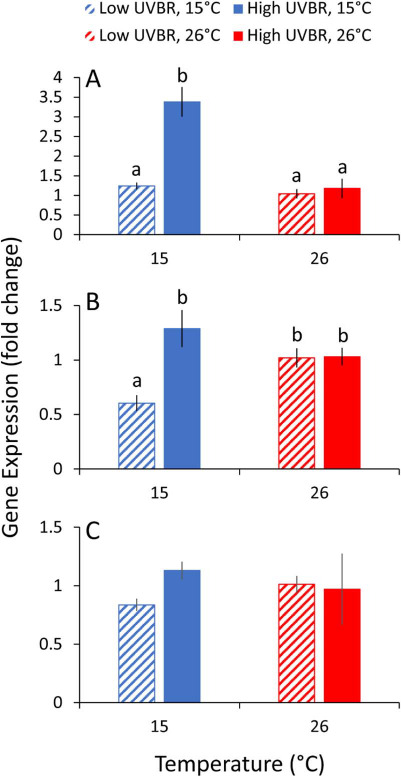
Effect of 4 weeks of exposure to high (solid bars) and low (patterned bars) UVBR in combination with low (blue bars) and high (red bars) temperature on expression of (**A**) CPD photolyase, (**B**) 6-4 photolyase and (**C**) glucocorticoid nuclear receptor in whole-body *L. peronii* larva tissue, displayed as the fold change compared to the low UVBR, high temperature treatment (*n* = 5 to 6 per treatment; Experiment 1). Data are presented as means ± standard error. Lowercase letters above bars denote significant differences between treatments. Where no letters are presented, no significant differences were detected between treatments

There was a significant interaction between elevated UVBR levels and temperature on CPD photolyase (*CPD-phr*) gene expression levels (*t*_19_ = −2.499, *P* = 0.022; Fig. 6A). High UVBR exposure had no effect relative to low UVBR on *CPD-phr* expression at 26°C (*χ*^2^_[1]_ = 0.025, *P* = 1), but led to a 3-fold higher expression at 15°C (*χ*^2^_[1]_ = 14.347, *P* < 0.001). A similar interactive effect between low temperature and high UVBR exposure was also present for *6-4phr* photolyase expression (*t*_19_ = −2.62, *P* = 0.017; Fig. 6B). Despite the absence of an effect of high UVBR at 26°C (*χ*^2^_[1]_ = 0.001, *P* = 1), larvae exposed to high UVBR at 15°C had more than double the expression of *6-4phr* compared to larvae that were not exposed to high UVBR in the 15°C treatment (*χ*^2^_[1]_ = 14.598, *P* < 0.001). There was no significant interaction between UVBR and temperature on the expression of the glucocorticoid nuclear receptor (*t*_19_ = −1.939, *P* = 0.068; Fig. 6C), and no main effects of UVBR (*t*_20_ = −0.537, *P* = 0.597) or temperature (*t*_20_ = −0.306, *P* = 0.763) on expression of this gene.

### Leucocyte profiles

**Figure 7:**
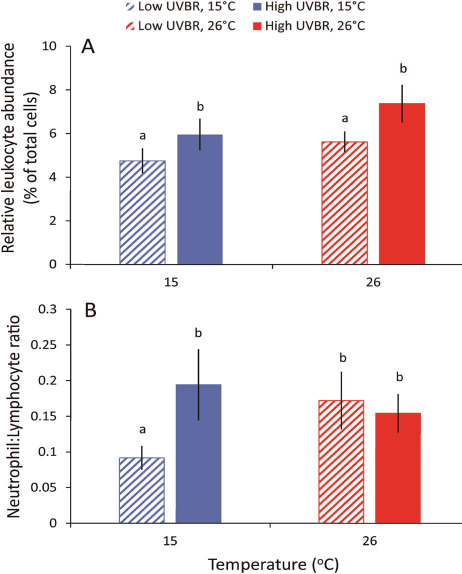
Effect of 4 weeks of exposure to high (solid bars) and low (patterned bars) UVBR in combination with either low (blue bars) or high (red bars) temperature on (**A**) the relative abundance of leucocytes (%), and (**B**) the neutrophil to lymphocyte ratios in *L. peronii* larval blood (*n* = 6–12 animals per treatment; Experiment 2). Data are presented as mean ± standard error. Lowercase letters above bars denote significant differences between treatments

Developmental stage had a significant effect on the leucocyte proportion of total cells (*Z*_1,37_ = 2.189, *P* = 0.029), whereby more developed larvae (up to Gosner stage 43) generally had a higher composition of leucocytes in their blood (up to 14% of total blood cells) than larvae at earlier developmental stages (down to Gosner stage 30), with a leucocyte percentage as low as 2%. Developmental effects on leucocyte abundance considered, larvae exposed to high UVBR had a greater proportion of leucocytes than larvae in the low UVBR treatments (*Z*_1,38_ = −2.853, *P* = 0.004), but there was no effect of temperature (*Z*_1,37_ = 0.631, *P* = 0.528; Fig. 7A).

There was a significant three-way interaction between temperature, UVBR and developmental stage on neutrophil to lymphocyte (N:L) ratios of larvae (*Z*_1,33_ = −2.214, *P* = 0.027; Fig. 7B). At 15°C, high UVBR increased the N:L ratio of larvae compared to low UVBR; however, this effect was dependent on developmental stage, meaning that such effects may not be consistent throughout the larval development period.

## Discussion

In this study, we demonstrated interactions between temperature and elevated UVBR on physiological and immunological traits of *L. peronii* larvae at the molecular, cellular and organismal levels. These results have implications for our understanding of how elevated levels of UVBR may contribute to ongoing amphibian declines. Consistent with earlier work on *L. peronii* (van Uitregt *et al*., 2007), high UVBR and low temperature interacted to influence larval survival, resulting in mortality rates greater than those predicted by the sum of their main effects. Interspecific variation in survival following UVBR exposure is largely determined by the efficacy of DNA repair pathways (UV sensitivity hypothesis; Blaustein *et al*., 1994; Schuch *et al*., 2015), suggesting that accumulation of DNA damage in individuals with poor DNA repair rates is the ultimate cause of death. In our study, ineffective DNA repair likely explains the inability of *L. peronii* larvae to cope with UVBR-exposure at low temperatures (Tevini, 1993; Pakker *et al*., 2000; MacFadyen *et al*., 2004; Morison *et al*., 2019). Lethal effects were apparent in our study even though UVBR levels were almost 10 times lower than those used by van Uitregt *et al*. (2007). This is likely a result of both the longer exposure period and lower temperature used in our study (15°C as opposed to 20°C; van Uitregt *et al*., 2007). Our results show that even relatively low intensity UVBR exposures can be lethal for *L. peronii* larvae when administered over a long time period. However, tadpoles in the low-temperature treatment received a 2-fold greater thermal change compared to tadpoles in the warm temperature treatment prior to UVBR exposure. That this difference also contributed to the responses of larvae to UVBR cannot be discounted. Despite this, our data support the hypothesis that the effects of elevated UVBR exposure can be exacerbated by prolonged exposure to low temperatures which may have implications for amphibians living at high altitude (Middleton *et al*., 2001; Blaustein *et al*., 2003). Also consistent with van Uitregt’s work, larvae exposed to low UVBR experienced higher mortality at high temperatures compared to larvae raised at low temperatures (van Uitregt *et al*., 2007), despite being well within the thermal tolerance of *L. peronii* larvae (Wilson and Franklin, 1999). The reason for this result is unknown, and no study to our knowledge has investigated the mechanistic basis for this effect.

There was no effect of elevated UVBR exposure (compared to low UVBR) on photolyase gene expression in the high temperature treatments. We suggest that constitutive expression levels of photolyase enzymes at high temperatures in *L. peronii* larvae are sufficient to cope with the DNA damage induced by the elevated UVBR treatment. At low temperature, however, we observed a 2–3-fold increase in expression of the two major photolyase genes (*CDP phr* and *6-4 phr*) in larvae exposed to high UVBR relative to low UVBR. Upregulation of DNA repair genes is generally triggered by the accumulation of DNA damage in epithelial cells (Schwarz and Schwarz, 2011; Damiani and Ullrich, 2016). Elevated photolyase gene expression in low-temperature, high UVBR-exposed larvae suggests that low temperatures enhanced the accumulation of UVBR-associated DNA damage possibly because of the thermal sensitivity of photolyase DNA repair, which is evident in this species (Morison *et al*., 2019). Given that DNA damage in epidermal cells is a known molecular trigger for the release of immunosuppressive cytokines such as interleukin-4 and 10 in mammals (Kripke *et al*., 1992; Schwarz and Schwarz, 2011; Damiani and Ullrich, 2016), we expected that the high level of molecular-level damage likely imposed by the high UVBR/low-temperature treatment combination would result in immunosuppression in larvae.

In addition to UVBR-induced DNA damage triggering immunosuppression, UVBR can also influence immune function through the production of ROS (Yang *et al*., 2013). In *L. peronii* larvae, exposure to high UVBR for 2 weeks induced a significant increase in TAC relative to low UVBR. This increase in TAC is likely a response to cope with additional UV-induced ROS production which is consistent with past work on this species (Kern *et al*., 2015) and in fish (Kazerouni *et al*., 2016a). After 4 weeks of exposure to high UVBR, there was no longer any difference in TAC between UVBR treatments. We suspect that the change in exposure regime midway through the experimental period, from 4 h of UVBR per day to 2 h per day, is responsible for this change in TAC between Weeks 2 and 4; however, this is temporally confounded and could be an effect of larval development rather than a treatment effect. There was no main effect of treatment temperature on TAC activity, suggesting that the thermal sensitivity of antioxidant enzymes in the high-temperature treatment was sufficient to counteract additional metabolic ROS production, without requiring an increase in antioxidant enzyme abundance (Kong *et al*., 2012; Meng *et al*., 2014; Kazerouni *et al*., 2016b).

Exposure to elevated UVBR at low temperature significantly reduced the growth and metabolic rate of *L. peronii* tadpoles compared to low UVBR. Independently, both low temperatures and high UVBR can impede growth in amphibian larvae (van Uitregt *et al*., 2007), albeit through different mechanisms. While low temperatures directly affect metabolic rate (e.g. Belden and Blaustein, 2002; Romansic *et al*., 2009; Jones *et al*., 2015), UVBR can reduce activity levels and foraging (Alton *et al*., 2012). Responding to UVBR is also energetically costly (Alton *et al*., 2011, 2012). In our study, larvae exposed to elevated UVBR at high temperature had an ~ 20% increase in energy expenditure relative to larvae exposed to high temperatures and low UVBR. In contrast, the effects of elevated UVBR at low temperatures on larval growth and development rates appeared to be antagonistic. A reduction in activity levels in high UVBR, low-temperature environments may impair energy acquisition, or UVBR may inhibit metabolic pathways or damage mitochondria directly, thus reducing the rate of cellular respiration in the organism (Cramp *et al*., 2014). Although this manifested most obviously as reduced growth and developmental rates in *L. peronii*, the distribution of finite energy resources across competing energetically expensive processes (such as growth, DNA damage and repair, antioxidant production and/or melanin synthesis) may mean that different traits are differentially affected by UVBR exposure at low temperature. For example, energy may be diverted away from growth towards more immediately critical functions including increased photolyase enzyme production and cellular repair (Sancar and Tang, 1993; MacFadyen *et al*., 2004). Immune function can also be impaired by investment in competing energetically demanding processes such as growth and reproduction (Demas *et al*., 2012). Maintaining a functionally responsive immune system and mounting an immune response to a pathogen challenge are energetically costly and require resources that could be allocated to other processes (i.e. growth; Demas *et al*., 2012).

Our results did not support the hypothesis that potential immunosuppressive effects of low temperature and high UVBR exposure would manifest as reduced leucocyte proportion of total cells in larvae, as observed elsewhere (Raffel *et al*., 2006; Ceccato *et al*., 2016; Franco-Belussi *et al*., 2018). Rather, larvae exposed to high UVBR for 4 weeks had a higher relative abundance of leucocytes than larvae in the low UVBR treatments, which likely reflects a chronic inflammatory immune response (McMahon *et al*., 2014). Elevated leucocyte counts are a systemic marker of UV-induced chronic inflammatory processes in mammals (Uluckan and Wagner, 2016). Inflammation is critical for normal immune responses against infection and promotes the regeneration of damaged host tissues (Tang and Wang, 2016). The elevated leucocyte proportion of total cells in tadpoles exposed to high UVBR may reflect a systemic inflammatory response that serves to repair damaged UVBR-exposed tissues. Importantly, chronic inflammatory processes are energetically costly (Demas *et al*., 2012) and may have contributed to the observed trade-offs with larval growth. The possibility that higher leucocyte proportions reflect a decrease in circulating erythrocytes and not an increase in leucocytes, or some combination of both (e.g. Sayed, 2016), cannot be discounted. Although we did not observe an obvious immunosuppressive phenotype (i.e. reduced leucocyte proportion of total cells) of *L. peronii* larvae exposed chronically to UVBR, assessment of leucocyte counts (by volume of blood) in the early stages of exposure may have yielded different results. Further research is also needed to determine if UVBR-induced DNA damage and metabolic compromise affects other immune function metrics and/or if immunosuppressive effects carry over into later life-history stages.

In addition to total white blood cell counts, the ratio between the number of circulating neutrophils to lymphocytes (N:L ratio) can provide useful information on the impacts of UVBR exposure on chronic inflammation and stress levels. Elevated N:L ratios are typically used in medical settings as markers for chronic inflammatory conditions such as cardiovascular disease, diabetes, renal disease and cancer (e.g. Ambatipudi *et al*., 2018; Bao *et al*., 2018; Tonyali *et al*., 2018). N:L ratios are also associated with stress levels in vertebrates including amphibians, because stress tends to increase the number of circulating neutrophils while reducing the number of lymphocytes in the blood (Davis *et al*., 2008; Davis and Maney, 2018). At 15°C, the N:L ratio was twice as high in *L. peronii* larvae exposed to high UVBR compared to those in the low UVBR treatment, suggesting that exposure to high UVBR at low temperature is a more stressful environment and caused a chronic inflammatory response. Although we were unable to measure plasma glucocorticoid levels directly in *L. peronii* larvae because of their small size, we found no evidence to suggest that glucocorticoid receptor gene expression patterns changed in response to UVBR exposure. This may be due to disparity in the responses of glucocorticoid levels and leucocyte profiles in response to long-term, chronic stress. While the glucocorticoid response often wanes during a period of chronic or repeated stress, N:L ratios tend to remain elevated, making them potentially a more practical measure of chronic stress (Davis and Maney, 2018). Alternatively, the non-specificity of the whole-body tissue samples used for analysis of glucocorticoid receptor gene expression and small sample size may also explain why no differences in expression were detected, if tissue-specific changes were present. The dependency of our results on developmental stage means that interactive effects of elevated UVBR and low temperature may not be consistent throughout all of the larval development period. This three-way interaction between UVBR, temperature and development on the N:L ratio may have occurred because glucocorticoids are also important neuroendocrine signalling molecules that play a central role in larval amphibian development (Denver, 2009). Further research is needed to assess whether chronic exposure to elevated UVBR at low temperatures is physiologically stressful and if so how this influences chronic inflammatory processes and developmental progression in larval amphibians.

This is the first study to demonstrate complex interactions between low temperature and low levels of chronic UVBR on integrated measures of survival, growth, energy expenditure and indices of immune function in amphibian larvae. The results support the hypothesis that elevated UVBR may contribute to amphibian population declines in cooler climates and at higher altitudes, both directly and indirectly through a range of sub-lethal effects. Our findings demonstrate the importance of considering the effects of environmental stressors in combination, since the effects of one stressor may only be manifested in the presence of another stressor. Further research into how UVBR and temperature interactions shape host–pathogen interactions are important for our understanding of spatial and temporal disease dynamics and amphibian population declines so that effective conservation plans can be implemented.

## Funding

This work was supported by Australian Research Council Discovery grants to CEF [DP140102773 & DP190102152].

## Supplementary Material

Supplementary_Figure_1_coaa002
