# Supplementary figures and images for: Effects of ultraviolet-B radiation on physiology, immune function and survival is dependent on temperature: implications for amphibian declines
Source: Conserv Physiol. 2020 Feb 11;8(1):coaa002. doi: 10.1093/conphys/coaa002 (PMC7245394; doi:10.1093/conphys/coaa002)

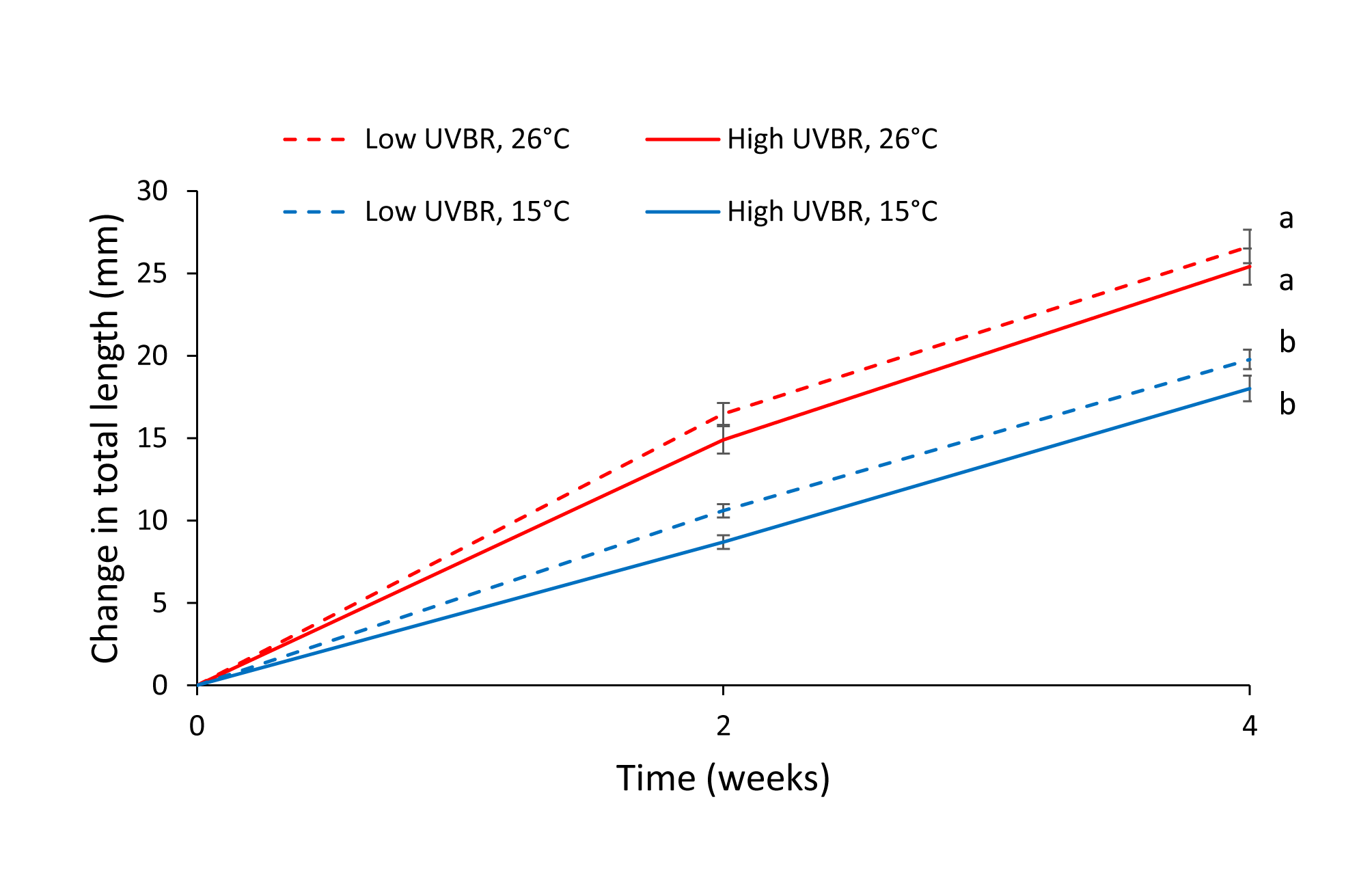

Supplement: Supplementary_Figure_1_coaa002 [file supplementary_figure_1_coaa002.zip › Supplementary_Figure_1_coaa002.tif]
